# A Comparative Genome-Wide Analysis of the R2R3-MYB Gene Family Among Four *Gossypium* Species and Their Sequence Variation and Association With Fiber Quality Traits in an Interspecific *G. hirsutum* × *G. barbadense* Population

**DOI:** 10.3389/fgene.2019.00741

**Published:** 2019-08-15

**Authors:** Nuohan Wang, Qiang Ma, Jianjiang Ma, Wenfeng Pei, Guoyuan Liu, Yupeng Cui, Man Wu, Xinshan Zang, Jinfa Zhang, Shuxun Yu, Lingjian Ma, Jiwen Yu

**Affiliations:** ^1^College of Agronomy, Northwest A&F University, Yangling, China; ^2^State Key Laboratory of Cotton Biology, Cotton Institute of the Chinese Academy of Agricultural Sciences, Key Laboratory of Cotton Genetic Improvement, Ministry of Agriculture, Anyang, China; ^3^Department of Plant and Environmental Sciences, New Mexico State University, Las Cruces, NM, United States

**Keywords:** genome-wide analysis, R2R3-MYB, genome-wide co-localization, fiber quality trait QTL hotspots, gene expression

## Abstract

Cotton (*Gossypium* spp.) is the most important natural fiber crop in the world. The R2R3-MYB gene family is a large gene family involved in many plant functions including cotton fiber development. Although previous studies have reported its phylogenetic relationships, gene structures, and expression patterns in tetraploid *G. hirsutum* and diploid *G. raimondii*, little is known about the sequence variation of the members between *G. hirsutum* and *G. barbadense* and their involvement in the natural quantitative variation in fiber quality and yield. In this study, a comprehensive genome-wide comparative analysis was performed among the four *Gossypium* species using whole genome sequences, i.e., tetraploid *G. hirsutum* (AD1) and *G. barbadense* (AD2) as well as their likely ancestral diploid extants *G. raimondii* (D5) and *G. arboreum* (A2), leading to the identification of 406, 393, 216, and 213 R2R3-MYB genes, respectively. To elucidate whether the R2R3-MYB genes are genetically associated with fiber quality traits, 86 R2R3-MYB genes were co-localized with quantitative trait loci (QTL) hotspots for fiber quality and yield, including 42 genes localized within the fiber length QTL hotspots, in interspecific *G. hirsutum* × *G. barbadense* populations. There were 20 interspecific nonsynonymous single-nucleotide polymorphism (SNP) sites between the two tetraploid cultivated species, of which 16 developed from 11 R2R3-MYB genes were significantly correlated with fiber quality and yield in a backcross inbred population (BIL) of *G. hirsutum* × *G. barbadense* in at least one of the four field tests. Taken together, these results indicate that the sequence variation in these 11 R2R3-MYB genes is associated with the natural variation (i.e., QTL) in fiber quality and yield. Moreover, the functional SNPs of five R2R3-MYB allele pairs from the AD1 and AD2 genomes were significantly correlated with the gene expression related to fiber quality in fiber development. The results will be useful in further elucidating the role of the R2R3-MYB genes during fiber development.

## Introduction

The MYB (myeloblastosis) transcription factors are widespread, and they comprise the most functionally diverse family of transcription factors in plants. They are characterized by a conserved MYB DNA-binding domain, generally comprising up to four imperfect repeats, each forming a helix-turn-helix (HTH) structure of approximately 53 amino acid residues. In plants, MYB proteins are classified into four subfamilies depending on the number of imperfect repeats in the MYB DNA-binding domain (one, two, three, or four), i.e., 1R-, R2R3-, 3R-, and 4R-MYB (Rosinski and Atchley, 1998; Jin and Martin, 1999). While there are only a few genes in the other three subfamilies, the R2R3-MYB family, which contains two imperfect repeats, is the predominant MYB family in plants.

In plants, R2R3-MYB transcription factors are the focus of numerous studies and have been shown to function in diverse plant-specific processes, including secondary metabolism, hormonal signaling, cell cycle control, cell fate determination, and responses to biotic and abiotic stresses (Paz-Ares et al., 1987; Wang et al., 2004). For example, *AtMYB11*, *AtMYB12*, and *AtMYB111*, which encode three *Arabidopsis* R2R3-MYB proteins, control the biosynthesis of flavonol (Stracke et al., 2007), and *AtMYB123* and *FaMYB9* play important roles in proanthocyanidin biosynthesis in *Arabidopsis* and *Fragaria*, respectively (Schaart et al., 2013). Moreover, R2R3-MYB transcription factors participate in mediating plant hormone actions and responses to biotic and abiotic stresses. In *Arabidopsis*, for example, *AtMYB96* mediates abscisic acid signaling during the drought stress response (Seo et al., 2009), and two chicory R2R3-MYB transcription factors, namely, CiMYB5 and CiMYB3, respond to abiotic stress and hormonal cues by regulating the expression of fructan 1-exohydrolase expression (Wei et al., 2017). In addition, several R2R3-MYB genes have been reported to function in pathogen defense, including two R2R3-MYB genes (*AtMYB30* and *AtBOS1*) in *Arabidopsis* and *PacMYBA*, which encode a R2R3-MYB protein in sweet cherry (Vailleau et al., 2002; Mengiste et al., 2003; Shen et al., 2017). Importantly, several types of R2R3-MYB proteins are also involved in the development and determination of cell fate, especially plant trichome development involving *Arabidopsis* leaf trichomes and cotton fibers (all unicellular epidermal hairs) (Kirik et al., 2005; Gonzalez et al., 2009; Li et al., 2009b). For example, in *Arabidopsis*, a regulatory complex with ENHANCER OF GLABRA3 (EGL3) and TRANSPARENT TESTA GLABRA1 (TTG1) is combined with an R2R3-MYB protein (GL1, AtMYB23 or AtMYB5) to form a regulatory complex to influence trichome initiation in leaves or the outer epidermal cells of the seed coat (Kirik et al., 2005; Gonzalez et al., 2009; Li et al., 2009b).

Cotton (*Gossypium* spp.) is the largest renewable source of textile materials, and the fiber is a differentiated single cell derived from ovules through four distinct and overlapping growth phases: initiation, elongation (primary cell wall synthesis), secondary cell wall synthesis, and maturation (Basra and Malik, 1984). The first two stages are known to determine the crucial traits of cotton ﬁber quality, such as fiber length (FL) and lint percentage (LP) (Ramsey and Berlin, 1976a; Ramsey and Berlin, 1976b). There is solid evidence that R2R3-MYB transcription factors are involved in the fiber development network. Previous results have suggested that two clades of R2R3-MYB transcription factors are associated with the first two processes (Wan et al., 2016). The first clade is the MYBMIXTA-like (MML) subgroup. In *Arabidopsis*, the MIXTA clade comprises subgroup 9 (S9), the members of which are known to affect the regulation of cellular differentiation, particularly in the formation of trichomes, root hairs, and conical cells (Baumann et al., 2007; Gilding and Marks, 2010). In cotton, previous studies *via* RNAi and ectopic overexpression confirmed that *GhMYB25* (*GhMML7*) and *GhMYB25-like* (*GhMML3_A12*) are involved in cotton fiber initiation and elongation (Machado et al., 2009; Walford et al., 2011), which are homologous to *AmMIXTA* (a MIXTA gene isolated from *Antirrhinum majus*) and regulate the epidermal conical cells (Perez-Rodriguez et al., 2005). Furthermore, a new *GhMYB25-like* (*GhMML3_A12*) regulation mechanism in fiber development was proposed in a recent study (Wan et al., 2016); namely, small RNA-mediated silencing of the *GhMYB25-like* (*GhMML3_A12*) gene contributes to the dominant mutant *N_1_* naked seed (i.e., fuzzless) trait in *G. hirsutum* (Wan et al., 2016). *GhMML4_D12*, another lint fiber development gene (*Li_3_*) cloned by the map-based cloning strategy, is involved in the switch to lint fiber development (Wu et al., 2018). Another clade of R2R3-MYB proteins are homologous to *AtMYB0/GLABRA1* and *AtMYB66/WEREWOLF*, which belong to subgroup 15 (S15) and are involved in epidermal patterning, trichome production, and root hair formation *via* the MYB-bHLH-WD40 protein complex in *Arabidopsis* (Stracke et al., 2001; Zhang et al., 2003). The cotton R2R3-MYBs orthologous genes corresponding to these characterized *Arabidopsis GLABRA1-like* members include *GaMYB2*, *GhMYB2*, *GbMYB2*, and *GhMYB109*, all of which show preferential expression levels in the fiber initiation and elongation stages (Suo et al., 2003; Wang et al., 2004; Guan et al., 2011; Huang et al., 2013). The silencing of *GhMYB109* results in a phenotype with a shorter fiber length in its transgenic cotton plants, indicating that this gene plays an important role in fiber elongation (FE) (Pu et al., 2008). The other three genes that are homologous to *GLABRA1* are *GaMYB2*, *GhMYB2*, and *GbMYB2*, which were isolated from *G. arboretum*, *G. hirsutum*, and *G. barbadense*, respectively, and are related to the development of the leaf trichome, fiber-like hairs and root hairs *via* ectopic overexpression in *Arabidopsis*. For example, in *Arabidopsis*, *GaMYB2* can rescue the phenotype of a *gl1* mutant (Wang et al., 2004), and *GhMYB2* induces seed to produce fiber-like hairs if overexpression occurs (Guan et al., 2011). Recently, Guan et al. (2014) showed that miR828 and miR858 regulate homologous *GhMYB2* functions in the development of the *Arabidopsis* trichome and cotton fiber (Guan et al., 2014). The third process (secondary cell wall synthesis) determines the fiber strength (FS) and fineness, which are also critical fiber quality traits. The *G. hirsutum* R2R3-MYB gene, *GhMYBL1*, is preferentially expressed in fibers at the secondary cell wall biosynthesis stage of fiber development. *GhMYBL1* overexpressing transgenic *Arabidopsis* overexpressing *GhMYBL1* has thickened vessels and interfascicular and xylary fiber cell walls in basal stems (Sun et al., 2015). However, there have been no reports on the involvement of R2R3-MYB genes in the fiber maturation process.

Biotic and abiotic stresses that threaten cotton fiber yield and quality are often encountered during the growing season, and several reports have suggested that R2R3-MYB proteins are involved in the responses to these stress. *GbMYB5*, which encode an R2R3-MYB protein in *G. barbadense*,**is positively involved with plant adaptive responses to drought stress, and virus-induced gene silencing of this gene compromises the tolerance of cotton plantlets to drought stress and reduces the post-recovery rewatering survival rate to 50% compared with the 90% survival rate in the wildtype (WT) (Chen et al., 2015). He et al. found that 52 *GrMYBs* and 46 *GaMYBs* in roots and leaves are differentially expressed under salt and drought stress treatment (He et al., 2016). *Verticillium* wilt (caused by the fungus *Verticillium* *dahliae*) is a highly destructive disease that leads to severe cotton yield losses worldwide and threatens cotton yield and fiber quality. Recently, a *V.* *dahliae*-responsive MYB gene, *GhMYB108*, which participates in the defense response through its interaction with the CaM-like protein GhCML11, was identified in *G. hirsutum* (Cheng et al., 2016).

R2R3-MYB proteins are one of the most abundant transcription factor families in cotton, and the genomic organization of the R2R3-MYB family has been studied in *G. hirsutum* and its potential ancestral diploid *G. raimondii* (D5), with 524 and 205 non-redundant cotton MYB genes identified, respectively (He et al., 2016; Salih et al., 2016). However, no comparative analyses have been performed among the four cotton species, including the two above, using sequenced genomes, so their sequence variation is currently unknown. Although the functional characterization of these putative R2R3-MYB genes has been identified with comprehensive genomic approaches that combine *in silico* bioinformatics analysis, none of the R2R3-MYB genes have been genetically studied for their involvement in the natural quantitative variation in fiber quality and yield.

In the present study, a genome-wide analysis of R2R3-MYB transcription factors was performed in four *Gossypium* species using whole genome sequences, i.e., tetraploid *G. hirsutum* (AD1) (Zhang et al., 2015) and *G. barbadense* (AD2) (Liu et al., 2015) as well as their potential ancestral diploids, *G. raimondii* (D5) (Paterson et al., 2012) and *G. arboreum* (A2) (Li et al., 2014; Du et al., 2018). *G. hirsutum* has higher yields and produces approximately 95% of the cotton fibers in the world, whereas *G. barbadense* produces much longer, stronger, and finer fibers. The present study focused on the sequence variation in terms of single-nucleotide polymorphisms (SNPs) and marker development of R2R3-MYB genes. The updated cotton quantitative trait loci (QTL) database (Version 2.3, www.cottonqtldb.org) (Said et al., 2015) was used to identify co-localization of R2R3-MYB genes with reported fiber quality and yield trait QTL from interspecific *G. hirsutum* × *G. barbadense* populations. Thus, recombinant inbred lines (RILs) and backcross inbred lines (BILs) developed from interspecific crosses between the two species can provide important genetic stocks to address the association of R2R3-MYB genes with fiber quality and yield.

## Materials and Methods

### Identification of the R2R3-MYB Gene Family in Four *Gossypium* Species

The genome sequences of *G. arboretum* (A2), *G. raimondii* (D5), *G. hirsutum* (AD1), and *G. barbadense* (AD2) were downloaded from the CottonGen database (https://www.cottongen.org/home), and putative R2R3-MYB genes were identified in the PFAM protein family database using HMMER software version 3.0 (Eddy, 2011), with the MYB-like DNA-binding domains (Pfam: PF00249) as the search query (Finn et al., 2013) and an initial threshold value of *E* ≤ 10^−10^. Clustal X v. 2.0.11 (http://www.clustal.org/) with default options was used for multiple sequence alignment. To identify potential relationships between the various R2R3-MYB gene family members, a neighbor-joining (NJ) phylogenetic tree was constructed *via* MEGA 5.0 (http://www.megasoftware.net/), and a multiple alignment analysis (ClustalW) and WebLogo 3 (http://weblogo.threeplusone.com/) (Doerks et al., 2002) were used to search the sequence logos for homologous domain amino acid sequences of R2 and R3 repeats. All R2R3-MYB genes in the four *Gossypium* genomes were mapped on the chromosome using the Circos-0.69-3 genome visualization tool (http://www.circos.ca/).

To clarify the evolutionary dynamics and selection pressures of R2R3-MYB genes in the four *Gossypium* species, the CODEML program from pamlX1.3.1 was used to estimate the ratio (ω) of the rate of nonsynonymous (dN) nucleotide substitutions to that of synonymous (dS) substitutions between the homologous protein-coding gene sequences of these R2R3-MYB orthologous gene pairs (Yang, 2007). An ω value significantly less than 1 indicates purifying selection (negative selection); ω > 1 indicates positive selection (diversifying selection); and ω = 1 suggests neutral evolution (Zhang et al., 2005).

### Plant Materials

Because the cultivated tetraploid cotton *G. barbadense* has superior fiber quality to that of *G. hirsutum*, introgression lines between the two species were used in this study. *G. hirsutum* CCRI 36 and *G. barbadense* cotton Hai 7124, which have different fiber quality and yield characteristics, were used to identify the putative interspecific SNPs *via* RNA-seq SNP calling. Ovules and fibers of CCRI 36 and Hai 7124 were excised from developing bolls at 5 days post-anthesis (DPA) for RNA-seq. In addition, developing fibers of the two cultivated species at different stages (10, 20, and 25 DPA) were used to perform the quantitative real-time (qRT) PCR analysis. Moreover, a BIL population (BC_1_F_7_) comprising 180 individual lines generated from the cross of (*G. hirsutum* CCRI 36 × *G. barbadense* Hai7124) × CCRI 36 and following seven generations of self-pollination was used to scan the gene type *via* high-resolution melting (HRM). The genomic DNA was extracted from young leaves of the 180 BILs and the two parents using a mini-prep method as described by Zhang and Stewart (Zhang and Stewart, 2000). The two parents and 180 BILs were grown in two replications using a randomized complete block design (RCBD) in Anyang, Henan, China in 2015 and 2016, Xinjiang, China in 2016, and Hainan, China in 2015−2016. The BILs and their parents were grown in the field under the same conditions, and the crop management practices and boll sampling followed local recommendations. All samples were flash-frozen in liquid nitrogen and stored at −80°C until use.

### Fiber Quality Measurements

The high volume instrument (HVI) 1000 fiber test system was used to determine the mature fiber qualities including fiber length (FL), fiber strength (FS), micronaire (MIC), fiber elongation (FE), fiber uniformity (FU), and short fiber content (SFC). The fiber quality tests were carried out using the same standard testing conditions at the Center for Cotton Fiber Quality Inspection and Testing under the supervision of the Chinese Ministry of Agriculture (Anyang, Henan, China) (Yu et al., 2013).

### Prediction and Co-Localization Analysis of R2R3-MYB Genes

In the present study, co-localization of the predicted *G. hirsutum* R2R3-MYB genes with fiber quality and yield QTL was used to screen for potential R2R3-MYB genes that may be involved in cotton fiber development. QTL were downloaded from CottonQTLdb (version 2.3, http://www.cottonqtldb.org) (Said et al., 2014; Said et al., 2015), and mapping of the GhR2R3-MYB genes was performed using MapChart (http://www.earthatlas.mapchart.com/) (Voorrips, 2002).

### Identification of SNPs for the Alleles of the R2R3-MYB Genes From *G. hirsutum* and *G. barbadense* and a Correlation Analysis With Fiber Traits

The RNA-seq dataset, which was retained in our laboratory, was used to identify SNPs for the R2R3-MYB genes. Total RNA was extracted from fibers and ovules collected from 5 DPA bolls of the two parents (CCRI 36 and Hai 7124) of the BILs, and cDNA libraries were constructed and sequenced using an HiSeq 2000 systems from Illumina. All reads were aligned to the TM-1 (*G. hirsutum*, Texas Marker-1) assembled by Nanjing Agricultural University (NAU). Two biological replicates from each sample were used for all RNA-seq experiments, and SNPs were called using Samtools (Li et al., 2009a) and Picard tools (http://broadinstitute.github.io/picard/).

Moreover, sequence variations in the predicted R2R3-MYB genes among the two sequenced tetraploid (2n = 4x = 52, AADD) species, *G. hirsutum* (TM-1 assembled by NAU and the Joint Genome Institute, JGI), and three *G. barbadense* were further analyzed. Three *G. barbadense* include 3-79 assembled by Huazhong Agricultural University (HAU) (Wang et al., 2019), Xinhai 21 assembled by HAU (Liu et al., 2015), and Hai 7124 assembled by Zhejiang University (ZJU) (Hu et al., 2019), respectively.

The HRM analysis was applied to confirm the putative SNPs of *G. hirsutum* and *G. barbadense* and do genotyping of the 180 BILs following the manufacturer’s protocol for the LightCycler^®^ 480 High Resolution Melting Master (Roche, Indianapolis, USA). Based on the different subgenomic sequences between CCRI 36 and Hai 7124, putative SNP primers for the R2R3-MYB genes in the corresponding sub-genomes were designed using Oligo 7 software to produce polymorphisms in the two parents of the BILs. The specificity of the primers was also determined by means of electronic-PCR amplification. A simple correlation analysis was performed using SPSS 12.0 software (IBM, New York, USA), and the primer pairs are shown in **Supplementary Table S1**.

### Analysis of 17 R2R3-MYB Genes in Two RNA-seq Datasets

To study the expression of the 17 *G. hirsutum* R2R3-MYB genes that co-localized with fiber trait and their alleles from *G. barbadense* at the fiber elongation and secondary cell wall synthesis stages, the transcriptional profiles of the 17 R2R3-MYB genes and their alleles were analyzed using information from the *G. hirsutum* TM-1 (Zhang et al., 2015) and *G. barbadense* cv Phytogen 800 (Tuttle et al., 2015) (SRP049330) transcriptome sequencing databases, which were downloaded from the Cotton Functional Genomics Database (https://cottonfgd.org/). The reads per kilobase per million mapped (RPKM) reads of TM-1 and Phytogen 800 fibers both involved three sampling time points: 10, 20, and 25 DPA for TM-1 fibers and 10, 21, and 28 DPA for Phytogen 800 fibers that included the time points of the two fiber development stages (fiber elongation and secondary cell wall synthesis). Transcriptome analyses were conducted with EvolView (http://www.evolgenius.info/evolview/).

### RNA Isolation and qRT-PCR Analysis

To study the differential expression of eight R2R3-MYB genes associated with fiber length and fiber strength during development between CCRI 36 and Hai 7124, total RNA was isolated from developing fibers at 10, 20, and 25 DPA, and each sample included three biological replicates. Based on the coding sequences of eight R2R3-MYB genes associated with fiber traits, gene-specific primers for qRT-PCR were designed with Oligo 7 software (**Supplementary Table S1**), and an RNA Prep Pure Plant kit (Tiangen, Beijing, China) was used to extract RNA. Then, 0.5 μg of purified total RNA was reverse-transcribed into cDNA using the Super Script First-Strand Synthesis System for qRT-PCR (PrimeScript, Takara, Dalian, China), following the manufacturer’s instructions. The gene transcript levels were calculated using the 2^−ΔΔCT^ method, and three biological replicates, each with three technical replicates, were evaluated. Analysis of variance followed by a t-test was performed using Microsoft Excel.

## Results

### Genome-Wide Identification of R2R3-MYB Genes in Four Sequenced *Gossypium* Genomes

**Figure 1:**
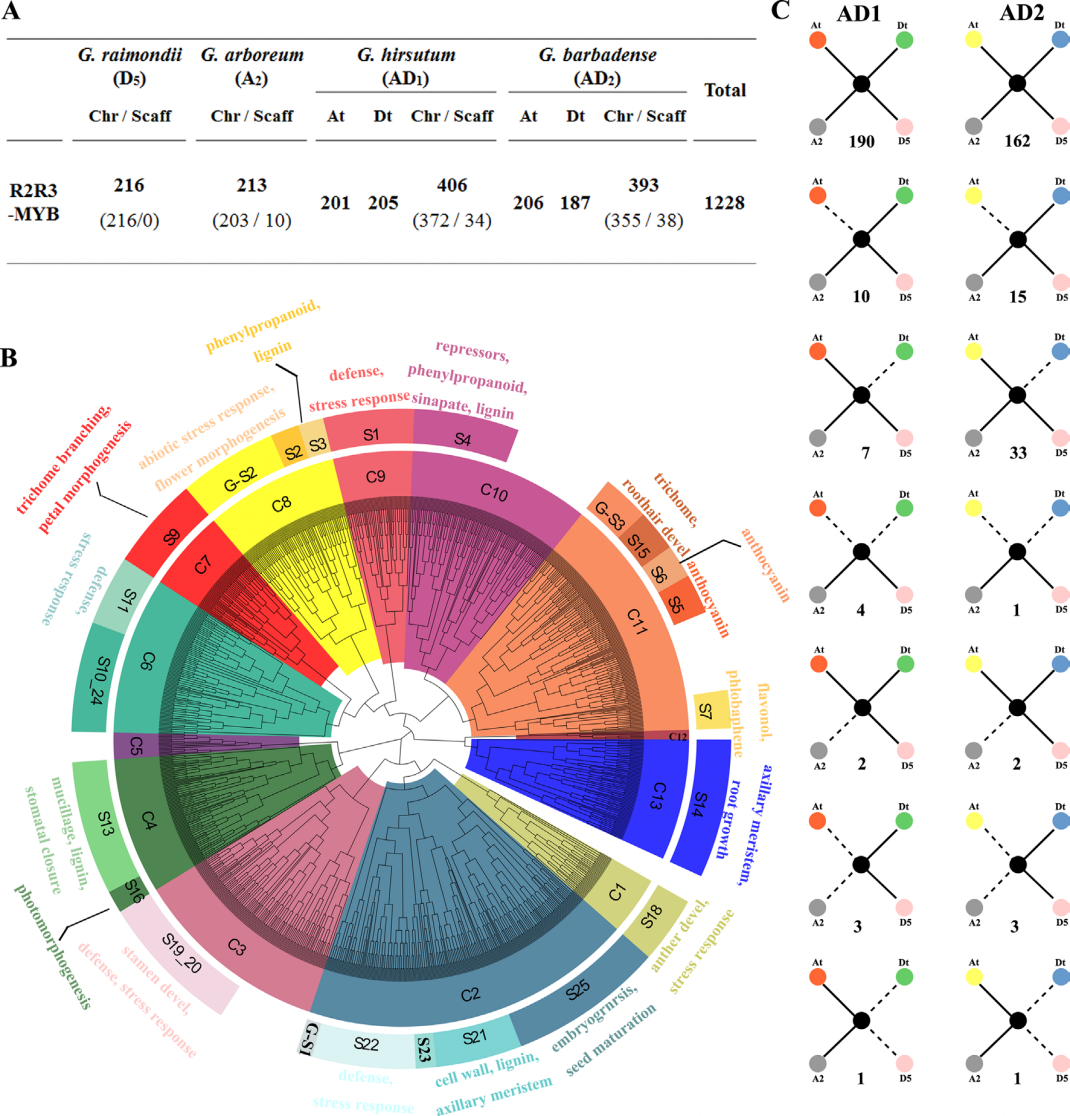
Phylogenetic and evolutionary analysis of the R2R3-MYB gene family in four *Gossypium* species. **(A)** The number of R2R3-MYB genes in the four *Gossypium* species. Chr and Scaff indicate the chromosome and scaffold. At and Dt indicate the A and D sub-genomes in tetraploid cotton *G. hirsutum* and *G. barbadense*, and ‘t’ indicates tetraploid. **(B)** Phylogenetic neighbor joining (NJ) tree (1,000 bootstraps) with R2R3-MYB proteins from four *Gossypium* species and *Arabidopsis*. Clades (and subgroups) are labeled with different colors, and the functional annotation of subgroup members is given. **(C)** Gene conservation scenarios and statistics for the four different genomes. The solid lines indicate observed genes, and the dotted lines indicate lost genes. The number of gene pairs found in the four different genomes that fit the specific model is provided below each graphic.

The whole genome sequence scaffolds from the ancestral diploids G. raimondii (D5) (Paterson et al., 2012) and G. arboreum (A2) (Li et al., 2014; Du et al., 2018) as well as their decedent tetraploids G. hirsutum (AD1) (Zhang et al., 2015) and G. barbadense (AD2) (Yuan et al., 2015) were used for a genome-wide search for R2R3-MYB genes in Gossypium. As a result, 216, 213, 406, and 393 R2R3-MYBs in the D5, A2, AD1, and AD2 genomes were identified, respectively (**Figure 1A**). A further phylogenetic analysis of all putative R2R3-MYBs from the four Gossypium species and 126 R2R3-MYBs of A. thaliana was performed to identify the evolutionary relationships involved in gene duplication and loss during the Gossypium evolution. Based on their predicted protein sequence similarity and phylogenetic tree analysis, the 1,228 R2R3-MYBs were divided into 13 clades (C1 to C13) (**Figure 1B**), where C2 and C12 are the largest and smallest clades with 214 and 6 cotton R2R3-MYBs, respectively (**Supplementary Table S2**). Based on the subgroup (S) categories from A. thaliana (Stracke et al., 2001; Dubos et al., 2010), the cotton R2R3-MYBs were classified into 22 subgroups that have been annotated in A. thaliana (**Figure 1B**). The present study also observed Gossypium-specific (G-S) clades (G-S1−G-S3) that were not clustered with A. thaliana (**Figure 1B**), indicating that these clades may have been lost in A. thaliana or acquired in Gossypium during the divergence from the last common ancestor. In the present study, MIXTA clade S9 (C7) and S15 (C11), characterized by AtMYB0/GLABRA1, are known to be involved in epidermal cell development, leading to the formation of trichomes and root hairs and include 55 and 22 R2R3-MYBs from four Gossypium species (**Supplementary Table S2**). As confirmed in previous studies, several cotton MIXTA and GLABRA1-like R2R3-MYB members are acquired during fiber development, such as GhMYB109 (Pu et al., 2008), GhMML3 (Wan et al., 2016), and GhMML4 (Wu et al., 2018). Therefore, the other members of S9 (C7) and S15 (C11) are the candidates for encoding the fiber development regulator.

In the present study, the gene loss and duplication of R2R3-MYB genes among the four *Gossypium* genomes were also analyzed, and seven specific models of gene duplication or loss events were found (**Figure 1C**). Extensive close orthologous relatives of R2R3-MYBs were identified in the four *Gossypium* species; namely, each GrR2R3-MYB or GaR2R3-MYB gene in each of the diploid species corresponded to two GhR2R3-MYB genes and two GbR2R3-MYB genes in the two tetraploid species that corresponded to the A and D sub-genomes, implying that whole genome duplication (WGD) was the main driving force of the R2R3-MYB gene expansion (**Figure 1C**). However, of the orthologous gene pairs, 17 and 48 were lost in *G. hirsutum* and *G. barbadense*, respectively, suggesting that *G. barbadense* had a higher rate of R2R3-MYB gene loss than *G. hirsutum*. Additionally, four gene pairs and one gene pair were lost from both the A and D sub-genomes of *G. hirsutum* and *G. barbadense*, respectively (**Figure 1C**), and four orthologous gene pairs were lost from *G. raimondii* during the formation of *G. hirsutum* and *G. barbadense* (**Figure 1C**). In addition, a total of eight gene pairs were lost from both the A or D sub-genome and their corresponding progenitors (**Figure 1C**).

**Figure 2:**
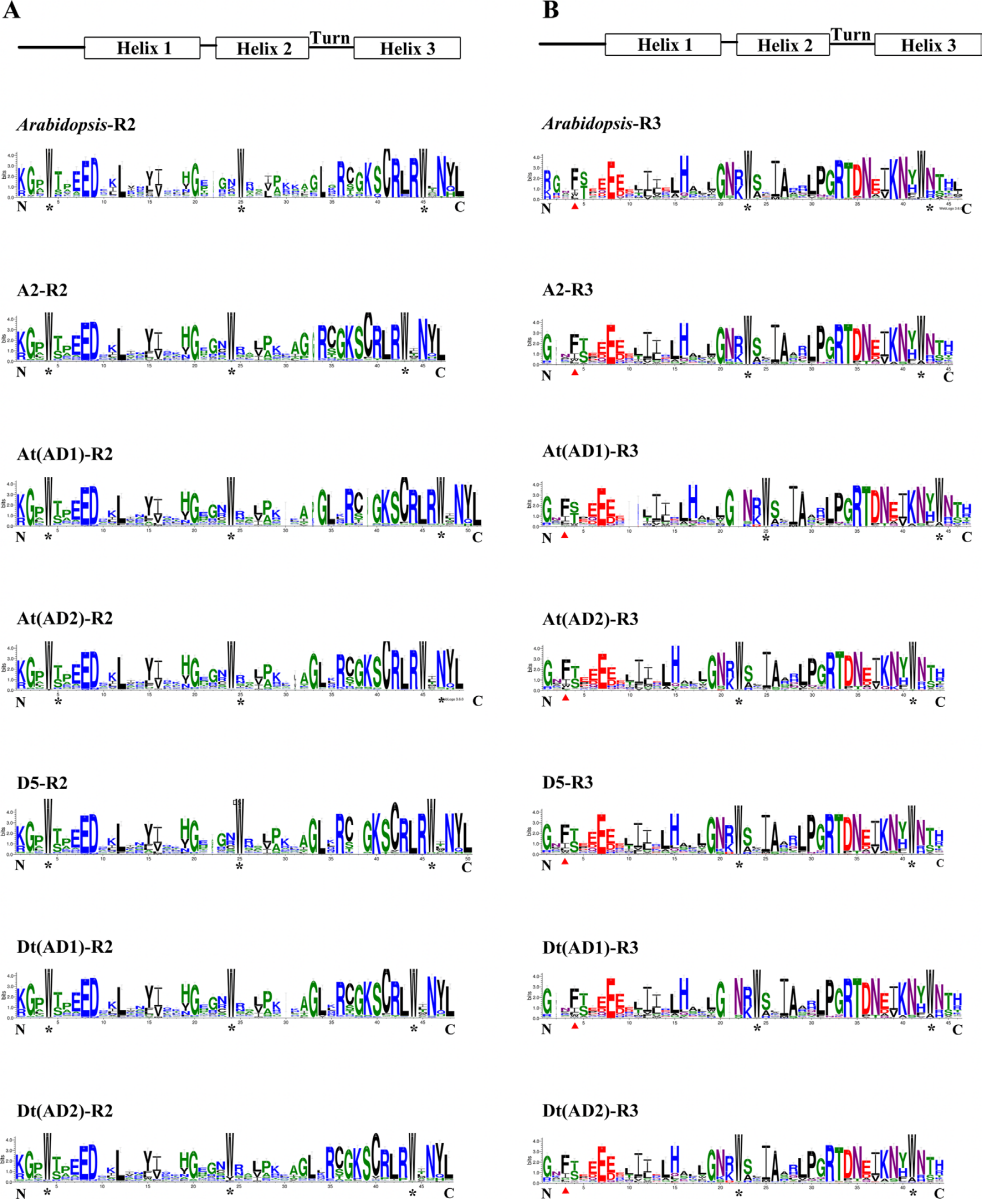
Comparison of DNA-binding domains of R2R3-MYB transcription factor proteins in four *Gossypium* species and *Arabidopsis*. **(A)** and **(B)** represent the R2 and R3 repeats, respectively. Highly conserved tryptophan amino acids are labeled with asterisks. Red triangles indicate the first conserved tryptophan residues in the R3 repeat, which were replaced.

To compare the features of the R2R3-MYB domain sequences among the four *Gossypium* genomes, the homologous domain amino acid sequences of 1228 R2R3-MYBs from the four *Gossypium* species and 126 from *A. thaliana* were aligned to produce the sequence logos. In general, the R2 and R3 repeats both covered approximately 53 amino acid residues with rare deletions or insertions (Stracke et al., 2014). **Figure 2** shows that the distribution of conserved amino acids of the R2 and R3 MYB domains of the four *Gossypium* species (including the A and D sub-genomes) were very similar to those of *A. thaliana*. A series of regularly spaced and highly conserved tryptophan (Trp, W) residues were observed in the R2 and R3 MYB repeats of all four *Gossypium* species (**Figure 2**), and in the R3 repeats, the first conserved W residues were replaced by phenylalanine (Phe, F) or less frequently by isoleucine (Ile, I), leucine (Leu, L), or tyrosine (Tyr, Y) (**Figure 2**). As shown in **Figure 2**, several highly conserved amino acid residues were mainly distributed in the turn and the third helix of the HTH motif, which is consistent with *A. thaliana*. Overall, there is an evolutionary conservation of R2R3-MYBs among the four *Gossypium* species.

**Figure 3:**
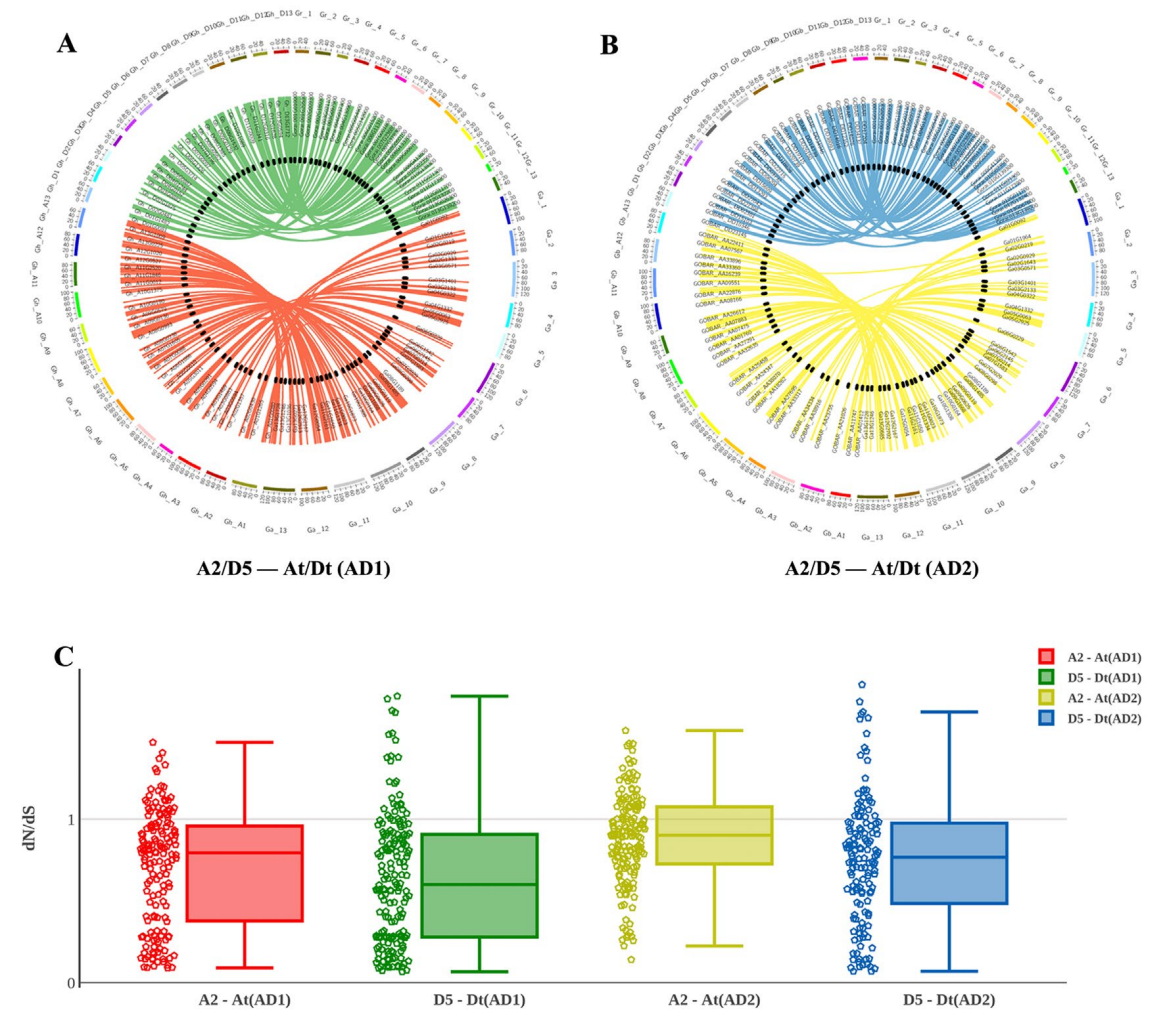
Chromosomal and codon substitution rate distribution between two sub-genomes of the two tetraploid cotton and their progenitors. **(A)** Chromosomal distribution in two sub-genomes of *G. hirsutum* and their progenitors. Red lines link homologous R2R3-MYBs between the A2 and At (AD1) chromosomes. Green lines link homologous R2R3-MYBs between D5 and Dt (AD1). **(B)** Chromosomal distribution in two sub-genomes of *G. barbadense* and their progenitors. Yellow lines link homologous R2R3-MYBs between A2 and At (AD2) chromosomes. Blue lines link homologous R2R3-MYBs between D5 and Dt (AD2). **(C)** The comparison of the dN/dS distribution among sub-genomes between their corresponding progenitor genomes.

After integrating the chromosomes of the sequenced cotton genomes, the 1228 R2R3-MYB candidate genes were mapped onto chromosomes or scaffolds, showing that the R2R3-MYB family members were unevenly distributed among chromosomes. In total, 1,146 of the 1,228 R2R3-MYBs were assigned to chromosomes, while the remaining 82 R2R3-MYBs were located in unmapped scaffolds (**Figures 3A, B**). For example, of the 372 GhR2R3-MYBs mapped to 26 chromosomes in *G. hirsutum*, 27 genes were mapped on chromosome 11 (chr11/A11), while chromosome 2 (chr2/A02) had only four genes. As shown by a line linking homologous R2R3-MYBs in **Figures 3A, B**, there are many syntenic gene blocks among the chromosomes of these four *Gossypium* species. Moreover, the present study compared the nonsynonymous (dN) and synonymous (dS) substitution rates (ω) of the 1228 R2R3-MYB genes between the sequenced progenitor genomes (A2 and D5) and the two sub-genomes of *G. hirsutum* and *G. barbadense* to explore the molecular evolutionary properties, and the results are shown in **Figure 3C**. In the A sub-genome and D sub-genome of *G. hirsutum* and *G. barbadense*, the ω values of most of the R2R3-MYB orthologous gene sets were below 1, suggesting that purifying selection occurred in the R2R3-MYB gene family during the evolution of diploid to allotetraploid. A few of gene sets (with ω > 1) experienced a positive selection, suggesting that these genes may play a key role in the evolution of allotetraploid *G. hirsutum* and *G. barbadense*. Interestingly, the R2R3-MYB gene family in the A sub-genome might have evolved faster than that in the D sub-genome both in *G. hirsutum* and *G. barbadense*, suggesting that asymmetric evolution occurred in the R2R3-MYB gene family for the two sub-genomes (**Figure 3C**). Furthermore, the R2R3-MYB genes in *G. barbadense* might have evolved faster than those in *G. hirsutum* in both the A and D sub-genomes, compared with their corresponding progenitor genomes (**Figure 3C**).

### Co-Localization of R2R3-MYB Genes With QTL for Fiber Quality and Yield

To determine whether any of the *G. hirsutum* R2R3-MYBs are genetically associated with fiber quality and yield, a genome-wide co-localization analysis was first performed for all genes coding for R2R3-MYB transcription factors in the sequenced genome TM-1 chromosomes A01 to A13 (or c1 to c13) and D01 to D13 (or c14 to c26) of the sequenced genome with QTL for fiber quality and yield in the interspecific *G. hirsutum* × *G. barbadense* populations downloaded from CottonQTLdb (Version 2.3, www.cottonqtldb.org). The fiber quality traits were fiber length (FL), fiber elongation (FE), fiber uniformity (FU), fiber strength (FS), lint percentage (LP), and micronaire (MIC). The fiber yield traits included percent fiber (PF), seed cotton yield (SCY), lint yield (LY), lint percentage (LP), and lint index (LI) (**Supplementary Table S3**). As a result, 48 fiber trait QTL hotspots (containing at least four QTL for the same trait within a 20-cM region (Said et al., 2013; Said et al., 2014; Said et al., 2015) were found, including 46 fiber quality trait QTL hotspots and two LP QTL hotspots distributed on different chromosomes (**Supplementary Table S4**). The 46 fiber quality trait QTL hotspots include 3 FE QTL hotspots, 16 FL QTL hotspots, 3 FS QTL hotspots, 4 FU QTL hotspots, and 20 MIC QTL hotspots that were distributed unevenly among chromosomes and mostly located on chromosomes A01/c1 (6) and D05/c19 (6) (**Supplementary Table S4**).

**Figure 4:**
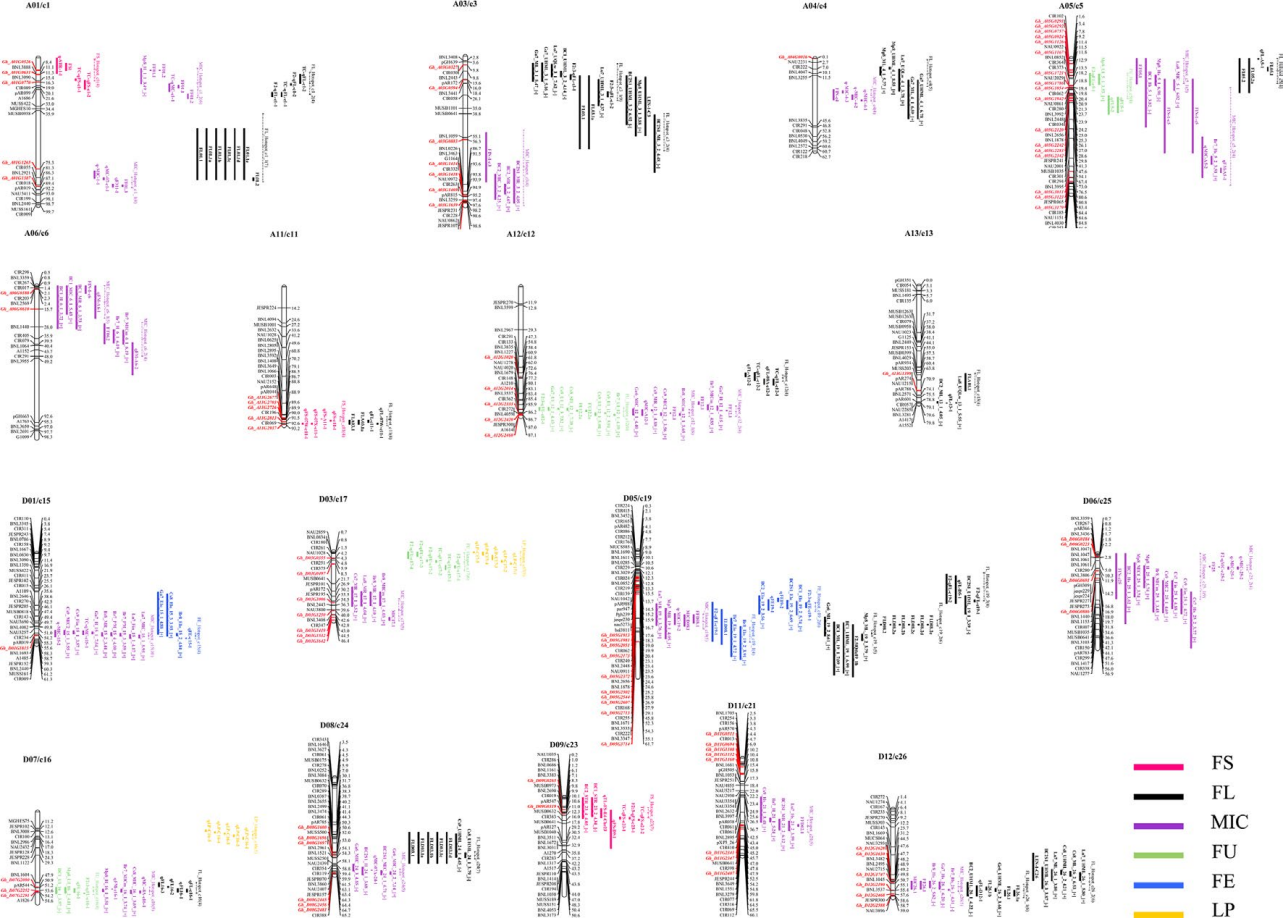
A genome-wide co-localization analysis of all R2R3-MYB genes in the sequenced genome TM-1 chromosomes with quantitative trait loci (QTL) hotspots for fiber trait in interspecific *G. hirsutum* × *G. barbadense* populations. The dotted line indicates the segment of the hotspot on the chromosomes.

The genome-wide co-localization analysis results indicate that 86 R2R3-MYB genes were localized within the fiber quality trait hotspots. Of the co-localized R2R3-MYB genes, 42 were localized within the FL hotspots. On chromosome D05/c19, which possessed the most fiber quality QTL hotspots among the chromosomes, 10 R2R3-MYB genes were localized within the hotspots (**Figure 4**). Some overlapping segments existed among different or in the same trait QTL hotspots within a 20-cM region, and the genes localized in the overlapping segments may be involved in the development of different traits. For example, *Gh_A05G1126*, an R2R3-MYB gene located on chromosome A05/c5, was co-localized with three fiber quality hotspots (i.e., FL_Hotspot_c5, FU_Hotspot_c5, and MIC_Hotspot_c5_1) (**Figure 4**).

### Sequence Variation in R2R3-MYB Genes Between *G. hirsutum* and *G. barbadense*

**Figure 5:**
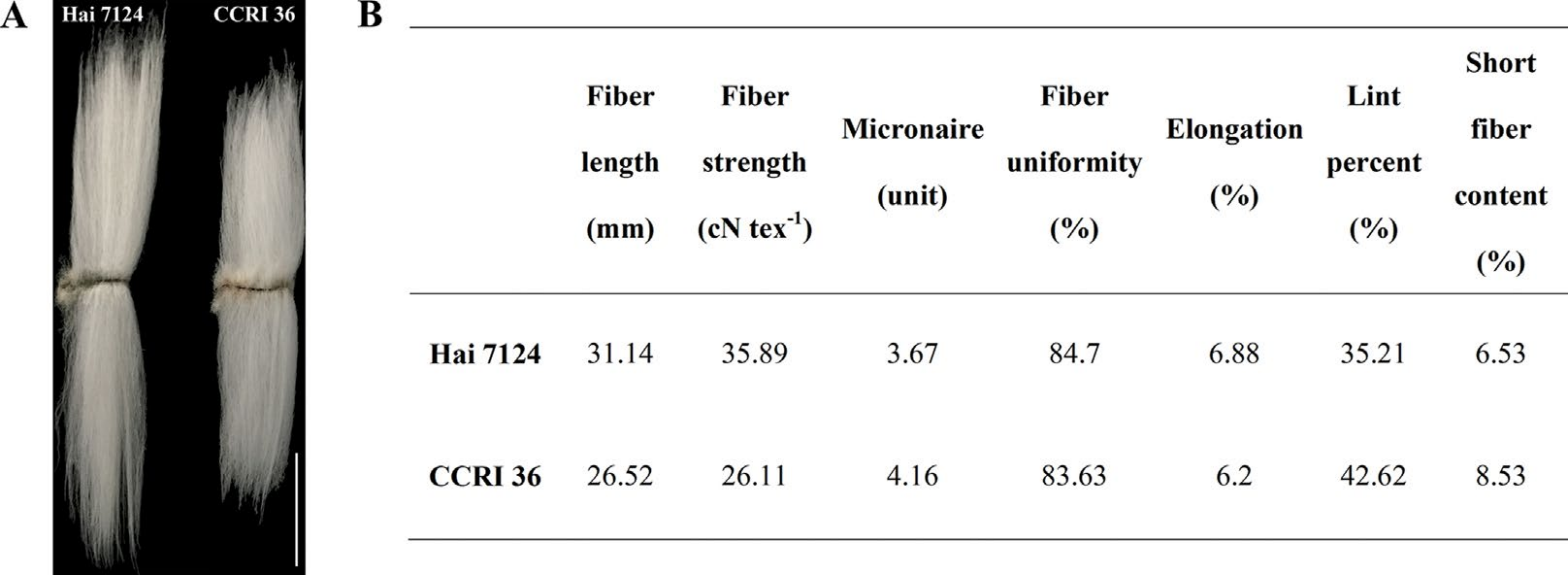
Fiber quality characteristics of Hai 7124 and CCRI 36. **(A)** Comparisons of mature fiber length of Hai 7124 and CCRI 36. Bar = 1.5 cm. **(B)** The mature fiber qualities were determined by the high volume instrument (HVI) 1000 fiber test system.

Because CCRI 36 and Hai 7124 differed in fiber quality and yield (**Figure 5**), the RNA-seq datasets of the two cultivated species were used to identify putative interspecific SNPs in the R2R3-MYB genes. Compared with the sequenced TM-1 genome (Zhang et al., 2015), 42 SNPs were identified in the cDNA sequences of 21 R2R3-MYB genes co-localized with fiber quality and yield trait QTL hotspots (**Supplementary Table S5**). Moreover, 27 nonsynonymous SNP sites in 17 R2R3-MYB gene sequences were detected between CCRI 36 and Hai 7124 (**Table 1**).

**Table 1:** The nonsynonymous single-nucleotide polymorphism (SNP) of the genes localized within the fiber quality quantitative trait loci (QTL) hotspots.

Gene ID	Chromosome_SNP_Position	TM-1_NAU	TM-1_JGI	CCRI 36	Hai 7124	Hai 7124_ZJU	Xinhai 21_NAU	3-79_HAU	SNP type
Gh_A01G1265	A01_SNP_75279139	C	C	C	A	A	A	A	Interspecific
Gh_A03G0883	A03_SNP_56268582	T	T	T	C	C	C	C	Interspecific
	A03_SNP_56268668	C	C	C	A	A	A	A	Interspecific
	A03_SNP_56269274	G	G	G	C	C	C	C	Interspecific
Gh_A03G1469	A03_SNP_94860485	T	T	T	C	C	C	C	Interspecific
Gh_A05G1126	A05_SNP_11408439	T	T	T	G	G	G	G	Interspecific
Gh_A05G2120	A05_SNP_24158570	C	C	C	T	T	T	T	Interspecific
Gh_A05G2342	A05_SNP_28567212	A	A	A	G	G	G	G	Interspecific
Gh_A05G3179	A05_SNP_83357615	G	G	G	A	A	A	A	Interspecific
	A05_SNP_83357788	C	C	C	G	G	G	G	Interspecific
	A05_SNP_83358199	A	A	A	G	G	G	G	Interspecific
Gh_A12G1020	A12_SNP_61827377	A	A	A	G	G	G	G	Interspecific
Gh_A13G1399	A13_SNP_70850590	G	A	G	A	G	G	G	Interspecific
Gh_D03G1250	D03_SNP_39979124	G	G	G	T	T	T	T	Interspecific
Gh_D05G2713	D05_SNP_29061704	C	C	C	T	T	T	T	Interspecific
	D05_SNP_29062660	A	A	A	G	G	G	G	Interspecific
Gh_D06G0184	D06_SNP_1821446	G	G	A	G	G	G	G	CCRI 36-specific
	D06_SNP_1821486	A	A	T	A	A	A	A	CCRI 36-specific
	D06_SNP_1821996	T	T	G	T	T	T	T	CCRI 36-specific
Gh_D11G2341	D11_SNP_45220974	C	C	C	T	T	T	T	Interspecific
Gh_D11G2347	D11_SNP_45682284	C	C	C	T	T	T	T	Interspecific
Gh_D11G2407	D11_SNP_47937585	T	T	T	G	G	T	T	Hai 7124-specific
	D11_SNP_47937659	T	T	T	G	G	T	T	Hai 7124-specific
Gh_D12G1630	D12_SNP_47650374	C	C	C	T	T	C	C	Hai 7124-specific
	D12_SNP_47650460	A	A	A	C	C	A	A	Hai 7124-specific
Gh_D12G1747	D12_SNP_49762738	A	A	A	G	G	G	G	Interspecific
	D12_SNP_49763474	G	G	G	A	A	A	G	Interspecific

Sequence variations in the predicted R2R3-MYB genes among the two sequenced tetraploid species, *G. hirsutum* (TM-1) (Zhang et al., 2015) and *G. barbadense* (Xinhai 21 and 3-79) (Liu et al., 2015; Yuan et al., 2015), were further analyzed. In the 27 nonsynonymous SNPs, there were three CCRI 36-specific SNPs (D06_SNP_1821446, D06_SNP_1821486, and D06_SNP_1821996) in *Gh_D06G0184* and four Hai 7124-specific SNPs (D11_SNP_47937585 and D11_SNP_47937659 in *Gh_D11G2407*, and D12_SNP_47650374 and D12_SNP_47650460 in *Gh_D12G1630*) compared with *G. hirsutum* (TM-1) and *G. barbadense* (Hai 7124, Xinhai 21 and 3-79). In addition, 20 true (i.e., homologous) interspecific SNP sites in 14 R2R3-MYB genes were detected between the two cultivated species (*G. hirsutum* and *G. barbadense*) using the public data (including genome data of TM-1, Xinhai 21, 3-79, and Hai 7124) as well as the RNA-seq data from our laboratory (**Table 1**). The results show that most of the sequence variations were detected between homologous genes from the two cultivated species, but a few CCRI 36 or Hai 7124-specific SNPs were also predicted based on the RNA-seq data. As these SNPs are from the R2R3-MYB genes within the fiber trait QTL hotspot regions, the differences in fiber quality traits between the two cultivated species are likely related to the natural sequence variations in the 14 R2R3-MYB genes, which will be verified by the following correlation analysis between the R2R3-MYB gene markers and the fiber quality and yield.

### Association Analysis Between R2R3-MYB Gene SNP Markers and Fiber Quality and Yield

For the association analysis between the existence of the 27 nonsynonymous SNPs in the R2R3-MYB genes and fiber traits, primers were designed to amplify the fragments containing these SNPs using HRM analysis in a BIL population of 180 lines derived from a backcross between *G. hirsutum* CCRI 36 and *G. barbadense* Hai 7124. To examine the relationship between the SNPs and fiber quality and yield, a correlation analysis was performed in the BIL population subjected to four replicated field tests. The presence of markers for 11 R2R3-MYB gene (Hai 7124 alleles) were found to have significant correlations with the fiber quality traits or LP in at least one test in the BIL population, but 11 markers for six other MYB genes were not significantly associated with fiber quality traits or LP (**Table 2**). Three *G. hirsutum* CCRI 36-specific SNP makers and four *G. barbadense* Hai 7124-specific SNP markers were also subjected to the association analysis, and the three CCRI 36-specific SNP markers significantly negatively correlate with MIC (−0.215, *P* < 0.01) (**Table 2**). These results indicated that the allele for the involved gene from CCRI 36 is likely to have a negative effect on MIC. Unfortunately, the four Hai 7124 specific SNP markers that involved in two genes had no significant correlation with fiber quality traits in the BILs, even though they are co-localized with MIC and FL, respectively. Perhaps, there are other genes in these regions that may affect the fiber quality traits, exhibiting a correlation.

**Table 2:** The correlation coefficients between the presence of the Hai 7124 allele of markers and fiber quality and yield in the backcross inbred line population of CCRI 36 × Hai 7124 hybrid tested in four environments.

Gene ID	Alleles of *G. barbadense*	Gene name	Chromosome_SNP_position	Co-localization trait	Correlation analysis in the backcross inbred line population of CCRI 36 × Hai 7124			
					SNP associated loci trait/PCC/environment			Associated loci repeated in four environments
Gh_A01G1265	GOBAR_AA11747	MYB4	A01_SNP_75279139	FL	FS	.149*	15-16_Hainan	3
						.202**	16_Xinjiang	
						.231**	16_Anyang_Xiaotun	
					FM	.156*	16_Anyang_Xiaotun	1
					FE	.155*	16_Anyang_Xiaotun	1
					FU	.201*	15_Anyang	1
Gh_A03G0883	GOBAR_AA38916	MYB44	A03_SNP_56268582	FL/MIC	FU	–.154*	15-16_Hainan	1
			A03_SNP_56268668		FS	–.149*	15-16_Hainan	1
			A03_SNP_56269274		MIC	–.189*	15-16_Hainan	2
						–.194*	16_Anyang_Xiaotun	
Gh_A03G1469	GOBAR_AA34334	DIVARICATA	A03_SNP_94860485	MIC	MIC	.156*	15-16_Hainan	1
Gh_A05G1126	GOBAR_AA33190	MYB26	A05_SNP_11408439	FL/FU/MIC	nc			
Gh_A05G2120	GOBAR_AA08895	MYB330	A05_SNP_24158570	FU/MIC	SFC	.162*	16_Anyang_Xiaotun	1
					FS	.192*	16_Anyang_Xiaotun	1
Gh_A05G2342	GOBAR_AA17937	MYB6	A05_SNP_28567212	MIC	MIC	.204**	15-16_Hainan	2
						.176*	16_Xinjiang	
					FL	–.170*	15-16_Hainan	1
					FM	.149*	16_Xinjiang	1
					SFC	.149*	16_Anyang_Xiaotun	1
Gh_A05G3179	GOBAR_AA15212	MYB39 (MIXTA)	A05_SNP_83357615	MIC	nc			
			A05_SNP_83357788					
			A05_SNP_83358199					
Gh_A12G1020	GOBAR_AA24908	MYB5	A12_SNP_61827377	FL	FE	.180*	15_Anyang	1
					MIC	.165*	15-16_Hainan	1
Gh_A13G1399	GOBAR_AA04000	MYB306	A13_SNP_70850590	FL	FE	.158*	15-16_Hainan	1
					MIC	.181*	15_Anyang	1
Gh_D03G1250	GOBAR_DD27042	MYB48	D03_SNP_39979124	MIC	FL	.167*	15-16_Hainan	2
						.172*	16_Xinjiang	
					FS	.148*	15-16_Hainan	2
						.179*	16_Xinjiang	
					MIC	–.169*	16_Anyang_Xiaotun	1
Gh_D05G2713	GOBAR_DD19015	GAMYB	D05_SNP_29061704 D05_SNP_29062660	FL	FL	–.360**	15-16_Hainan	4
						–.290**	15_Anyang	
						–.260**	16_Xinjiang	
						–.309**	16_Anyang_Xiaotun	
					FE	–.247**	15_Anyang	3
						–.167*	16_Xinjiang	
						–.302**	16_Anyang_Xiaotun	
					FS	–.153*	15-16_Hainan	2
						–.170*	16_Anyang_Xiaotun	
					FU	–.230**	15-16_Hainan	3
						–.242**	15_Anyang	
						–.210**	16_Xinjiang	
					SFC	.249**	16_Xinjiang	2
						.187*	16_Anyang_Xiaotun	
Gh_D06G0184	GOBAR_DD21017	MYB44	D06_SNP_1821446	MIC	MIC	–.215**	16_Xinjiang	1
			D06_SNP_1821486					
			D06_SNP_1821996					
Gh_D11G2341	GOBAR_DD00056	MYB306	D11_SNP_45220974	MIC	nc			
Gh_D11G2347	GOBAR_DD14016	MYB44	D11_SNP_45682284	MIC	MIC	–.160*	15-16_Hainan	4
						–.202**	15_Anyang	
						–.181*	16_Xinjiang	
						–.225**	16_Anyang_Xiaotun	
					LP	–.153*	15-16_Hainan	1
					FM	–.173*	16_Anyang_Xiaotun	1
Gh_D11G2407	Gb-NAU-D11 47621936-47620704	MYB5	D11_SNP_47937585	MIC	nc			
			D11_SNP_47937659					
Gh_D12G1630	GOBAR_DD14076	GhMML4D_12 (*Li3*)	D12_SNP_47650460	FL	nc			
			D12_SNP_47650374					
Gh_D12G1747	GOBAR_DD10004	MYB306	D12_SNP_49762738	FL/MIC	nc			
			D12_SNP_49763474					

** and * indicate correlations at the 0.01 and 0.05 levels of significance, respectively. nc, no correlation; PCC, Pearson correlation coefficient; Chromosome_SNP_Position, the position of the SNP in the TM-1 genome.

Furthermore, several SNP markers that were stably correlated with fiber quality traits were identified through the multiple field tests. For example, *Gh_D05G2713* encodes a gibberellin-regulated R2R3-MYB transcription factor, named GhGAMYB, and in the BIL population, the presence of the marker for *GbGAMYB* (Hai 7124 allele) was found to be significantly negatively correlated with FL in all four tests, with FE in three tests, with FS in two tests, and with FU in three tests. The correlation of the *GbGAMYB* gene marker with SFC was significantly positive in two tests (**Table 2**). As SFC and other fiber traits were negatively correlated, the opposite effects of the marker on SFC and other fiber traits were not unexpected. However, the opposite was true in *G. hirsutum*. Therefore, the allele for the *GAMYB* gene originated from *G. hirsutum* CCRI 36 improved fiber length, strength, and uniformity, but increased short fiber content. Moreover, the presence of the marker for *GOBAR_AA11747* (Hai 7124 allele) was found to be significantly positively correlated with FS in three tests (**Table 2**), suggesting that *GOBAR_AA11747* may promote fiber strength.

Taken together, these results suggest that the sequence variations in these 11 R2R3-MYB genes are associated with the natural variation in fiber quality and yield. It is generally known that *G. barbadense* has superior fiber quality to that of *G. hirsutum* (Zhang et al., 2014), but not all R2R3-MYB gene (Hai 7124 allele) markers had a significant positive correlation with fiber quality in the present study, because the expression of these genes at the transcription level is likely involved in the natural variation in fiber quality between *G. hirsutum* and *G. barbadense* (see next section).

### Expression Pattern of 17 Selected R2R3-MYB Genes in *G. hirsutum* and *G. barbadense*

**Figure 6:**
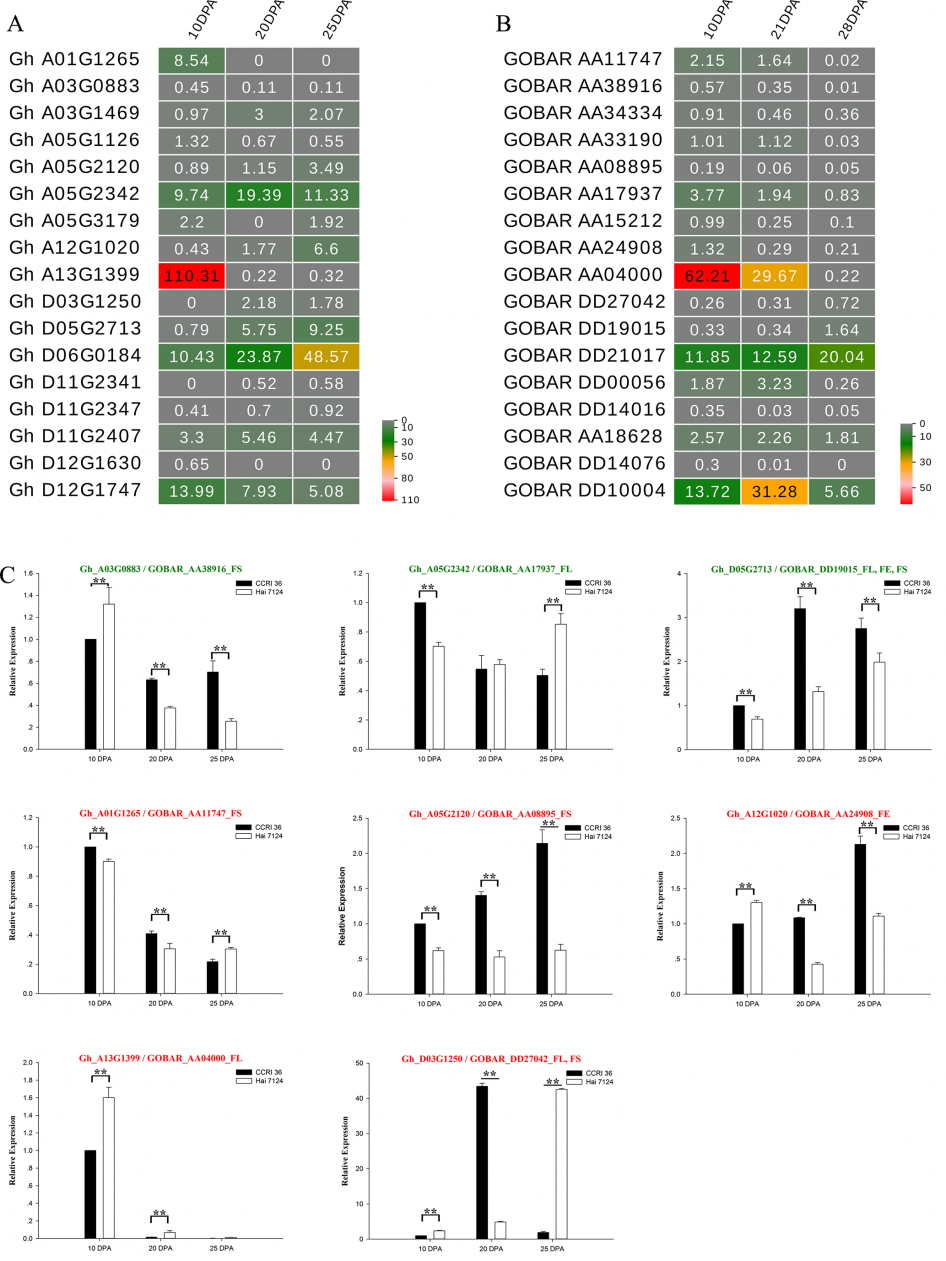
Expression patterns of 17 R2R3-MYBs in *G. hirsutum* and *G. barbadense* fibers. **(A)** A heat map showing transcript levels of 17 R2R3-MYBs in fibers at three stages (10, 20, and 25 DPA; shown above each lane) in TM-1. **(B)** Transcript levels of 17 R2R3-MYBs in fibers at three stages (10, 21, and 28 DPA; shown above each lane) in *G. barbadense* cv Phytogen 800. **(C)** Expression patterns of eight R2R3-MYBs in fibers of CCRI 36 and Hai 7124 at three stages (10, 20, and 25 DPA). The text highlighted in green and red shows the genes with negative and positive correlation, respectively. The SNP associated loci traits followed the gene ID. ** and * indicate correlation at the 0.01 and 0.05 levels of significance, respectively.

To understand the general pattern of gene expression for 17 *G. hirsutum* R2R3-MYB genes that were co-localized with fiber traits and their alleles from *G. barbadense* at the fiber elongation and secondary cell wall synthesis stages, the fragments per kilobase per million (FPKM) reads in the fiber at the two stages were compared between *G. hirsutum* TM-1 (Zhang et al., 2015) and *G. barbadense* cv Phytogen 800 from RNA-seq (Tuttle et al., 2015). Nearly all of these R2R3-MYB genes had transcript abundances at the two stages (**Figures 6A, B**), and the expression patterns of several R2R3-MYB homologous genes from the AD1 and AD2 genomes exhibited similar trends (upregulation or downregulation) from the fiber elongation stage to the secondary cell wall synthesis stage. The expression levels of three *Gh*-*Gb* gene pairs, namely, *Gh_A01G1265*-*GOBAR_AA11747*, *Gh_A03G0883*-*GOBAR_AA38916*, and *Gh_A13G1399*-*GOBAR_AA04000*, were decreased (**Figures 6A, B**). Interestingly, *GOBAR_AA04000* maintained a high expression level at 10 DPA and 21 DPA but rapidly decreased at 28 DPA, but the sharp decrease in expression of its *G. hirsutum* gene allele, *Gh_A13G1399*, occurred earlier during fiber development at 20 DPA (**Figures 6A, B**). This result indicated that the sequence variation in the coding regions and tightly linked regulatory regions of the alleles that originated from AD1 and AD2 is likely to lead to the variation in expression pattern at the early secondary cell wall synthesis stage, resulting in the difference in fiber strength between *G. hirsutum* and *G. barbadense*. In addition, the expression of two *Gh*-*Gb* gene pairs, namely, *Gh_D05G2713*-*GOBAR_DD19015* and *Gh_D06G0184*-*GOBAR_DD21017*, showed a tendency to increase (**Figures 6A, B**). Remarkably, the transcript level of *GOBAR_DD19015* exhibited a slight increase from the fiber elongation stage to the secondary cell wall synthesis stage (from 10 DPA to 28 DPA), while its *G. hirsutum* allele, *Gh_D05G2713*, sharply increased from 10 DPA to 20 DPA, and this level maintained to the late fiber secondary cell wall synthesis stage (25 DPA), indicating that the Upland genotype of this gene plays a more important role in fiber secondary cell wall synthesis than its *G. barbadense* counterpart. In addition, certain R2R3-MYB homologous gene pairs, such as *Gh_D12G1747* and *GOBAR_DD10004*, did not follow a hard and fast down- or up-regulation rule.

In cotton, the linkage disequilibrium (LD) decay distance is approximately 742.7 kb for all the accessions with variation among different genetic groups (Ma et al., 2018). So the variations in promoter sequence are in linkage disequilibrium with the functional SNP, which is in the coding region of a gene. Because the promoter sequence often directly affects RNA expression levels, LD makes the functional SNP to have a close relation with gene expression. To examine whether the sequence variation in R2R3-MYB genes between *G. hirsutum* and *G. barbadense* indirectly causes differences in the expression level, thus leading to functional differences, eight R2R3-MYB genes associated with fiber length, elongation, and strength (including three genes with negative correlations and five genes with positive correlations) were selected to further determine their expression levels in fibers at the elongation and secondary cell wall synthesis stages of CCRI 36 and Hai 7124 by qRT-PCR. Three points in time, namely, 10, 20, and 25 DPA, were investigated to emphasize the contrasts in fiber qualities between the two cultivated species.

Five typical allele pairs (i.e., *Gh_A03G0883*-*GOBAR_AA38916*, *Gh_D05G2713*-*GOBAR_DD19015*, *Gh_A05G2342*-*GOBAR_AA17937*, *Gh_A12G1020*-*GOBAR_AA24908*, and *Gh_A13G1399*-*GOBAR_AA04000*) showed an association between expression levels and functional SNPs (**Figure 6C**). If the presence of the Hai 7124 allele SNP markers negatively correlated with fiber length, elongation, or strength, the Hai 7124 allele exhibited a lower level of expression than the CCRI 36 allele for the three fiber quality traits at specific development times, and the reverse was true for a positive correlation. For example, the functional SNPs of *GOBAR_DD19015* (*GbGAMYB*) had consistent and significant negative correlations with FL, FE, and FS in at least two field tests, and they had lower transcript levels at 10, 20, and 25 DPA compared to those of *Gh_D05G2713* (*GhGAMYB*) (**Table 2** and **Figure 6C**). In addition, the significant positive correlation of the *GOBAR_AA04000* SNP markers with FL corresponded to the higher expression level at 10 DPA, which is the rapid fiber elongation stage (**Table 2** and **Figure 6C**). The three other allele pairs also displayed such a relationship at a specific time point. Taken together, these results indicated that these functional SNPs are likely to indirectly affect the RNA expression levels at specific fiber development stages and that the expression of these R2R3-MYB genes at the transcription level is likely involved in the natural variation of fiber length, elongation, and strength between *G. hirsutum* and *G. barbadense*. With regard to the remaining R2R3-MYB genes, no association was found among sequence variation, expression level, and phenotypic variation.

## Discussion

### The R2R3-MYB Gene Family in Four *Gossypium* Species

As an advantageous gene family in cotton fiber development, there is solid evidence that the R2R3-MYB members play important roles in the fiber initiation, elongation, and secondary cell wall synthesis stages (Suo et al., 2003; Wang et al., 2004; Machado et al., 2009; Bedon et al., 2014; Sun et al., 2015; Wan et al., 2016). In the present study, 1228 R2R3-MYB members were identified in the genomes of four *Gossypium* species, i.e., tetraploid *G. hirsutum* (AD1) and *G. barbadense* (AD2) and their potential ancestral diploids *G. raimondii* (D5) and *G. arboreum* (A2). The rate of the number of putative R2R3-MYBs in two tetraploid cotton species (AD1 and AD2) to two potential ancestral diploid cotton species (A2 and D5) is in good accordance with the proportion of the number of predicted genes in the sequenced genomes between tetraploid and diploids, as shown in **Figure 1A**, suggesting that the abundance of R2R3-MYBs in tetraploid cotton has expanded due to WGD. For example, the number of R2R3-MYBs in *G. hirsutum* is approximately 1.8 times to two diploid cotton species (*G. raimondii* and *G. arboretum*), consistent with the proportion between the numbers of predicted genes in the genome (*G. hirsutum* (70478)/*G. raimondii* (40960) ≈ 1.8 and *G. hirsutum* (70478)/*G. raimondii* (37505) ≈ 1.8) (Paterson et al., 2012; Zhang et al., 2015; Du et al., 2018). To understand the potential evolutionary relationships between the various R2R3-MYB gene family members among four *Gossypium* species, an NJ phylogenetic tree was constructed, and the putative protein sequences were divided into 13 subgroups. Consistent with previous studies that found that the allotetraploid genome experienced a higher frequency of genic sequence losses than the diploid genomes during polyploidization (Zhang et al., 2015), the present study observed the loss of more R2R3-MYBs in the two tetraploid cotton species instead of their diploid ancestors, suggesting a higher gene loss rate in *G. hirsutum* and *G. barbadense*.

To examine the driving force of gene evolution, the present study analyzed the nonsynonymous and synonymous substitution ratio (ω = dN/dS) of the orthologous gene sets. Most of the R2R3-MYB orthologous gene pairs had mainly evolved under the inﬂuence of purifying selection during *Gossypium* evolution. However, positive selection is generally difficult to detect because it often acts on a few sites and in a short period of evolutionary time, and the signal may be swamped by the ubiquitous negative selection. Furthermore, the orthologous gene sets from A2 and the A sub-genome had a more relaxed selection pressure than that from D5 and the D sub-genome, suggesting that asymmetric evolution occurred in the A and D sub-genomes of the R2R3-MYB genes. This result is consistent with the asymmetric evolution event of the whole genome, which tested 21,618 orthologous gene sets during the evolution of *Gossypium* (Zhang et al., 2015).

In this part of the study, the phylogenetic relationships involved in gene duplication and loss and the codon substitution rate distribution of R2R3-MYB family members were evaluated in four Gossypium species, and the results offered a useful framework for future research to understand the evolution of the R2R3-MYB gene family.

### Candidate Gene Identification Using a Genome-wide Co-Localization Analysis of an Advantageous Gene Family in Fiber Development

There are ∼45 diploid species and seven tetraploid species in the genus *Gossypium*, including four globally cultivated species (*G. hirsutum* L., *G. barbadense* L., *G. herbaceum* L., and *G. arboreum* L.) (Wendel and Cronn, 2003; Grover et al., 2014; Grover et al., 2015; Gallagher et al., 2017), of which two tetraploid cultivated species, *G. hirsutum* and *G. barbadense*, are grown commercially around the world for natural textiles. *G. hirsutum* has a high yield potential that accounts for >95% of the total global fiber production, but *G. barbadense* is renowned for having better fiber qualities (superior fiber strength, length, and fineness) (Zhang et al., 2014). In previous studies, extensive interspecific populations (crossing of *G. hirsutum* × *G. barbadense*) that combined the productivity of *G. hirsutum* with the fiber quality of *G. barbadense* were created by geneticists to leverage valuable genes for fiber qualities and yield improvement *via* QTL mapping. A total of 406 *G. hirsutum* R2R3-MYB genes were identified, so rapid and effective identification of candidate genes that are genetically associated with fiber quality and yield in advantageous gene families, such as R2R3-MYB, is critical.

In the present study, putative fiber development candidate R2R3-MYB genes were identified by a genome-wide co-localization analysis in an interspecific *G. hirsutum* × *G. barbadense* population, and the localization of the gene family members within fiber quality or yield trait QTL hotspot regions was investigated. Extensive fiber quality and yield trait QTL in *G. hirsutum* × *G. barbadense* populations (including 807 fiber quality traits QTL and 198 yield traits QTL) were downloaded from CottonQTLdb (Version 2.3), which contains 4,892 QTL from 156 publications. Subsequently, the QTL interval on the sequenced TM-1 genome was confirmed by its flanking marker sequence or primers, and 46 fiber quality trait QTL hotspots and two LP QTL hotspots were found. In the above QTL hotspots, 86 putative fiber development R2R3-MYB genes were identified and distributed on 18 chromosomes; these putative genes represented candidates for the QTL hotspots identified in the present study.

The putative candidate genes identified in the genome-wide co-localization analysis will require additional studies to confirm an association between their sequence variation and the natural variation (i.e., QTL) in fiber quality and yield. *G. hirsutum* CCRI 36 and *G. barbadense* cotton Hai 7124, which have different fiber quality and yield traits, were used to identify the putative interspecific SNPs *via* RNA-seq. Of the 86 putative fiber development R2R3-MYBs, 14 possessed nonsynonymous interspecific SNP sites in the gene sequences between the two cultivated species (*G. hirsutum* and *G. barbadense*). Subsequently, an advanced BIL population (BC_1_F_7_) comprised of 180 individual lines generated from the (CCRI 36 × Hai7124) × CCRI 36 cross was used in this study to confirm the relationship between the SNP markers and fiber quality and yield. The association analysis confirmed that the presence of the 11 R2R3-MYB gene nucleotide sequences with nonsynonymous SNP sites from *G. barbadense* were stably correlated with fiber qualities and lint percentage in the BIL population of *G. hirsutum* × *G. barbadense* in at least one test. Of the 16 SNP markers developed from the 11 R2R3-MYB genes, three SNPs, which are correlated with fiber qualities and present in two genes, were detected in all four field tests. Similar to traditional QTL mapping, the more field tests used for validation with, the more reliable the candidate genes were considered, and such reliable candidate genes were also detected in the present results. For instance, the presence of the *GbGAMYB* (Hai 7124 allele) markers were found to have a significant negative correlation with fiber length and fiber elongation in four and three tests, respectively, in the BIL population (**Table 2**). *GAMYB* encodes a gibberellin-induced R2R3-MYB transcription factor, and it has been reported to be a negative regulator of cell growth (Alonso-Peral et al., 2010). Overexpression of PtrMYB012, a poplar *GAMYB* homolog, in *Arabidopsi*s was shown to reduce the length of the petiole and stem (Kim et al., 2018), and in transgenic barley, the size of the anther was decreased by overexpression of the *HvGAMYB* gene (Murray et al., 2003). Therefore, the present results are consistent with those observed in poplar and barley, suggesting that the *GAMYB* allele originating from Hai 7124 may be involved in negative regulation during fiber elongation. In addition, *GhMML4_D12*, a lint fiber development gene (*Li_3_*) (Wu et al., 2018), was co-localized with a fiber length hotspots in the present study.

The great advantage of this method is that the increasing numbers of *Gossypium* population types (including intraspecific *G. hirsutum*, interspecific *G. hirsutum* × *G. barbadense*, and nature populations) created by breeders or geneticists will facilitate more QTL hotspot studies. Moreover, the genomes of the two cultivated tetraploid cotton species (AD1 and AD2 genomes) were sequenced and the genome-wide co-localization analysis of the advantageous gene family elucidated the interspecific differences in fiber quality and yield traits between AD1 and AD2. In the present study, the QTL hotspots of the interspecific *G. hirsutum* × *G. barbadense* population were focused on mining the candidate genes validated by introgression lines between the two species and there are a correspondingly vast number of QTL studies on an intraspecific *G. hirsutum* population in CottonQTLdb. Because the genetic variation in the diverse *G. hirsutum* germplasms and alleles involved in fiber quality is narrow, it is difficult to validate the putative candidate genes of a gene family based on one or several segregated populations of intraspecific *G. hirsutum*, which limits the application of genome-wide co-localization analysis in an intraspecific *G. hirsutum* population. Moreover, following the resequencing of additional core collections of *G. hirsutum*, it will be beneficial to test the results of the genome-wide co-localization analysis against the genome-wide association study (GWAS) data.

Overall, the present study provides an important foundation for further analysis to identify the valuable putative genes from an advantageous gene family for fiber development. Moreover, our results demonstrated a potential genetic engineering to the improve cotton quality and yield.

## Author Contributions

JZ and LM conceived the study, participated in its design, and drafted the manuscript. SY and JY directed the experiments. WP, JM, GL, and YC performed the field cultivation of cotton plants and ovules collection. NW and QM performed the experiments, and NW wrote the manuscript. JZ, MW and XZ revised the manuscript. All authors read and approved the final manuscript.

## Funding

This study was supported by the National Natural Science Foundation of China (grant no. 31621005), the National Key Research and Development Program of China (grant no. 2018YFD0100300 and 2016YFD0101400), and the National Research and Development Project of Transgenic Crops of China (grant no. 2016ZX08005005).

## Conflict of Interest Statement

The authors declare that the research was conducted in the absence of any commercial or financial relationships that could be construed as a potential conflict of interest.

The reviewer YY declared a shared affiliation, with no collaboration, with several of the authors, NW, QM, WP, GL, YC, MW, XZ, SY, and JY, to the handling editor at time of review.
